# 604. Safety and Efficacy of Oxandrolone vs Somatropin/Testosterone in Adult Patients with Severe Burns

**DOI:** 10.1093/jbcr/irag033.415

**Published:** 2026-04-06

**Authors:** Teana Terrell, Allison N Boyd, Lauren A Lautenslager, Leigh J Spera, Serena Dine, McKensie Hagen, Ashley E Thomas, Elaina Thomas, Todd A Walroth

**Affiliations:** Indiana University Health, Indianapolis, IN; Eskenazi Health, Indianapolis, IN; Eskenazi Health, Indianapolis, IN; Indiana University Health, Indianapolis, IN; Eskenazi Health, Indianapolis, IN; Indiana University Health, Indianapolis, IN; Eskenazi Health, Indianapolis, IN; Eskenazi Health, Indianapolis, IN; Eskenazi Health, Indianapolis, IN; Eskenazi Health, Indianapolis, IN

## Abstract

**Introduction:**

Historically oxandrolone, a testosterone analogue, was used to prevent muscle breakdown in severe burn patients and was shown to reduce length of stay (LOS) and increase weight gain. However, the Food and Drug Administration (FDA) removed this agent from the market in June 2023. Alternative therapies include testosterone, an anabolic steroid, and somatropin, a recombinant human growth hormone (rhGH). The purpose of this study was to compare the safety and efficacy of oxandrolone vs somatropin/testosterone in adult patients with severe burns.

**Methods:**

A single-center, retrospective, observational, cohort, non-inferiority study conducted at an American Burn Association (ABA)-accredited burn center. This study consisted of two treatment arms, oxandrolone and somatropin/testosterone. Data was collected from 11/28/2022-11/27/2023 in the oxandrolone group and 11/28/2023-11/30/2024 in the somatropin/testosterone group. The primary outcome was LOS and safety outcomes included incidence of hepatic dysfunction in patients who received oxandrolone or testosterone and hyperglycemia in patients who received somatropin.

**Results:**

The oxandrolone group (n = 20) and somatropin/testosterone group (n = 21) were well-matched with respect to age, total body surface area (TBSA), and type of burn. There was not a statistically significant difference in LOS for oxandrolone versus somatropin/testosterone (35 days versus 30 days, *p*=.787). Four (20%) of the oxandrolone patients required dose adjustments due to hepatic dysfunction versus two in the somatropin/testosterone group (*p*=.125) and hyperglycemia was reported in three (60%) of somatropin patients. Additionally, there was not a statistically significant difference between oxandrolone and somatropin/testosterone in secondary outcomes including mortality rate (15% versus 24%, *p*=.697), infection rate (70% versus 57%, *p*=.388), and change in weight (-2.6 pounds [lbs] versus -0.77 lbs, *p*=.598).

**Conclusions:**

Preliminary results support noninferiority of somatropin/testosterone to oxandrolone when assessing mortality rate, infection rate, weight loss, and LOS with no significant increase in safety concerns.

**Applicability of Research to Practice:**

This study is the first to our knowledge to compare oxandrolone to both testosterone and somatropin as alternatives. This study serves as an important step toward addressing the current treatment gap experienced by burn centers across the country, with plans to contribute this data to an ongoing multicenter analysis.

**Funding for the study:**

N/A.

